# Influence of contact map topology on RNA structure prediction

**DOI:** 10.1093/nar/gkaf1370

**Published:** 2025-12-17

**Authors:** Christian Faber, Utkarsh Upadhyay, Oskar Taubert, Alexander Schug

**Affiliations:** John von Neumann Institute for Computing, Jülich Supercomputing Centre, Forschungszentrum Jülich, D-52428 Jülich, Germany; John von Neumann Institute for Computing, Jülich Supercomputing Centre, Forschungszentrum Jülich, D-52428 Jülich, Germany; Scientific Computing Centre, Karlsruhe Institute of Technology, D-76344 Eggenstein-Leopoldshafen, Germany; John von Neumann Institute for Computing, Jülich Supercomputing Centre, Forschungszentrum Jülich, D-52428 Jülich, Germany; Faculty of Biology, University of Duisburg-Essen, D-45141 Essen, Germany

## Abstract

The available sequence data of RNA molecules have greatly increased in the past years. Unfortunately, while computational power is still under exponential growth, the computer prediction quality from sequence to final structure is still inferior to labour-intensive experimental work. Although a reliable end-to-end procedure has already been developed for proteins since Alphafold2, while its successor AlphaFold3 can also predict RNA, its confidence, in particular for novel sequences and folds, still appears limited. Another strategy entails two steps: (i) predicting potential contacts in the form of a contact map from evolutionary data; and (ii) simulating the molecule with a physical force field while using the contact map as restraint. However, the quality of the structure prediction crucially depends on the quality of the contact map. Until now, only the proportion of true positive contacts was considered as a quality characteristic. We propose to also include the distribution of these contacts, and have done so in our recent studies. We observed that the clustering of contacts, as is common for many artificial intelligence algorithms, has a negative impact on prediction quality. In contrast, a more distributed topology is beneficial. We have applied these findings from computer experiments to current algorithms and introduced a measure of distribution, the Gaussian score.

## Graphical Abstract

**Graphical Abstract:**
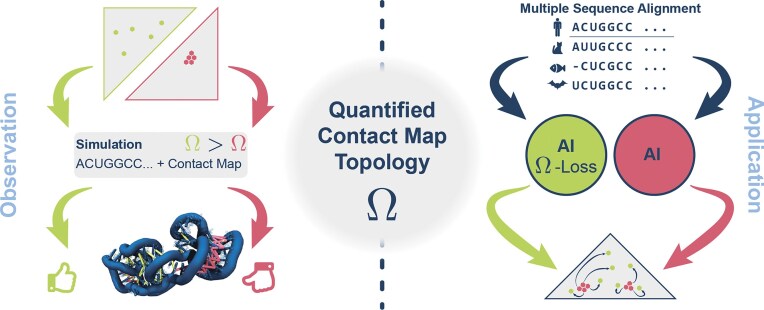

## Introduction

According to the central dogma of molecular biology, RNA functions as an intermediate product in protein biosynthesis [1]. In the years following this publication, however, more and more observations were made that RNA fulfils further functions. The influence of the structure of tRNA, for example, was already discussed in the 1980s [2], as was the structure of rRNA [3]. These RNA molecules, that do not code for a protein, are called non-coding RNAs (ncRNAs) [4]. Similar to proteins, the spatial structure of ncRNA is crucial for its ability to function successfully [5]. Therefore, the correct determination of the structure is of fundamental importance for the understanding of ncRNA, including evolutionary paths [6], virus genesis [7], and also the design of new drugs [8, 9]. The problem is that unlike sequencing, the experimental determination of the structure is a very labour-intensive task [10, 11]. Therefore, an *in silico* approach that requires less effort would be a major advance and achievement.

Immense progress has been made in the field of protein structure prediction in the last decade. Due to the large amount of available data, it was possible to train large language models and thus achieve an end-to-end prediction that comes close to experimental quality. The pioneering implementation of Google with AlphaFold [12] should be mentioned in this context. In the case of RNA structure prediction, such an assessment is rather problematic due to data limitations [13, 14]. To illustrate the difference in the data situation, we can look at the published Protein Data Bank (PDB) structures in the central database rcsb.org: for proteins, we have 196 400 structures and for RNA only 1890 (status: November 2024). As the end-to-end machine learning (ML) approaches are not yet fully developed, are there other possibilities?

**Figure 1.**
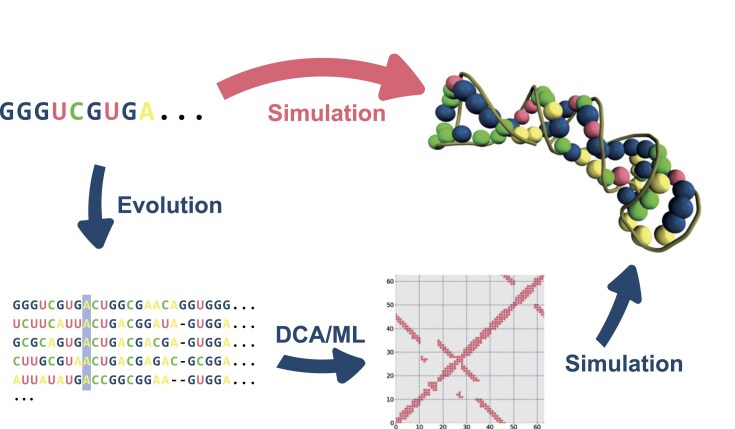
Schematic representation of the typical RNA structure prediction workflow. The upper route produces significantly worse results without the detour via contact map creation.

Various physics-based methods are available to determine the three-dimensional structure of RNA molecules. The most prominent representative for the structure folding, but also function determination of molecules is molecular dynamics (MD) simulation [15–17]. For example, it can be used to describe the full dynamics of RNA folding [18, 19]. However, while MD simulations are very accurate, when used in the context of structure prediction they require very large amounts of computing time, even when introducing biasing constraints [20, 21]. For this reason, simplified models are often used in practice for structure prediction, which can determine the stationary folding more quickly using Monte Carlo algorithms. Stationary folding is characterized by the fact that the molecules have reached a stationary state (or ensemble), i.e. their state (or ensemble) does not change further over time. Similarly, much effort has been put into judging the quality of RNA structure prediction [22]. We studied here how to achieve reliably good predictions with moderate computing resources. To achieve this, we use a workflow that has been successfully used in protein structure prediction since before AlphaFold. In the following, we will describe this process in a little more detail; for a better understanding, it is shown graphically in Fig. 1.

Two popular prediction software packages are the Monte Carlo-based SimRNA [23] and the fragment-based program Rosetta [24]. Both are capable of converting the initial sequence directly into a three-dimensional RNA structure. With them, the structure prediction quality can vary considerably with excellent predictions of low root mean square deviation (RMSD), e.g. 4plx, but also, for example, 3q3z only achieving an RMSD of ~29 Å in our computer experiments. Evolutionary data can support predictions by acting as helpful additional biasing constraints and considerably improve the quality of predictions [25, 26] by assuming that co-evolutionary mutational patterns are a result of spatial adjacency. To detect co-evolutionary signals, homologous sequences from other organisms are collected for the initial sequence $\lbrace a^0_i\rbrace _{i=1,...,L}$ with $a^0_i \in \lbrace A,U,C,G\rbrace$ and organized as multiple sequence alignment (MSA) $\mathcal {D} = \lbrace a_i^r| i=1,...,L\ \mathrm{and}\ r=1,...,M\rbrace$ with $a_i^r \in \lbrace A,U,C, G, -\rbrace$, $M$ the number of sequences. The co-evolution of different residues can be analysed with methods used in statistical physics, for instance mean field direct coupling analysis (DCA) [25, 27, 28], or with modern artificial intelligence (AI) algorithms [29–31]. These methods give us a binary mapping $\mathrm{CM}:\ [1:L] \times [1:L] \rightarrow \mathbb {Z}_2$, called a contact map, which indicates whether two residues $i,j$ are spatially adjacent [the nitrogen atoms (N1/N9) are ≤9.5 Å apart], i.e. form a contact. This definition of a contact goes back to one of the first papers on RNA contact maps and has since been frequently used in the literature. In contrast to proteins, where the $C_{\alpha }$ atom has established itself as a reference point, this is not so clear for RNA [32]. In simulations, these contacts can be used as restraints. The outcomes of these simulations are notably superior when evolutionary data are incorporated. Thus, the task is on the one hand to have a good data basis for the creation of MSAs and on the other hand to develop algorithms that provide as much information as possible from the MSA to the simulation software. In the past, the latter was often implemented using the direct coupling analysis method mentioned above. To measure the quality of the prediction, we often use the positive predicted value (PPV), which divides the number of correctly predicted contacts (true positives) by the total number (true positives + false positives). The bare DCA with mean field approximations achieves PPVs of ~50% [33–35]. The co-evolutionary analysis can also be carried out using ML algorithms. The PPVs of these algorithms reach values of up to 80% [29, 30], which is a remarkable increase. Due to the high PPV, one would assume an improved structure prediction by the simulation. However, detailed investigations were unable to confirm this expectation [30]. In fact, the increased PPV through a convolutional neural network (CNN) brought almost no improvement in prediction quality compared with the DCA-generated contact map. This result could have two causes. Firstly, the prediction quality could have an upper limit due to the simulation software, i.e. a further improvement of the restraints is unnecessary. On the other hand, the contact maps generated by AI algorithms could be of poorer quality, despite a high PPV. The AI algorithms are trained to achieve the best possible PPV, but the PPV is a massive dimension reduction of the prediction and ground truth. The PPV calculates a value from the contact map of the experimental structure and the contact map of the evolutionary analysis, i.e. $\mathrm{PPV}:\ \mathbb {Z}_2^{L\times L} \times \mathbb {Z}_2^{L\times L} \rightarrow [0,1]$. ML methods, like CNNs [36], suggest that contacts may be predicted in clusters wherever possible, therefore the PPV of such contact maps is still high, but information about smaller contact areas is lost. In this study we wished to investigate whether this effect of contact clusters is really responsible for the reduced performance in structure prediction and to introduce a mathematical measure to prevent this effect. This measure can be integrated into the objective function of ML algorithms to generate more diverse topologies of contact maps. To address these questions, we first present the software used and our test set in the Materials and methods. This is followed by an explanation of different methods to model various kinds of topologically different contact maps for our test set and an introduction of the new measure to gauge the diversity. We also describe the design of the computer experiments and how the influence of false contacts was investigated. Finally, the different applications are presented. In the Results and discussion section, the results of the computer experiments are discussed and the value of the new measure is shown directly in the applications. In the Conclusion, we summarize the most important findings again, but also address weaknesses and problems with the computer experiments carried out. Finally, we discuss possible continuations in modern AI algorithms.

**Figure 2.**
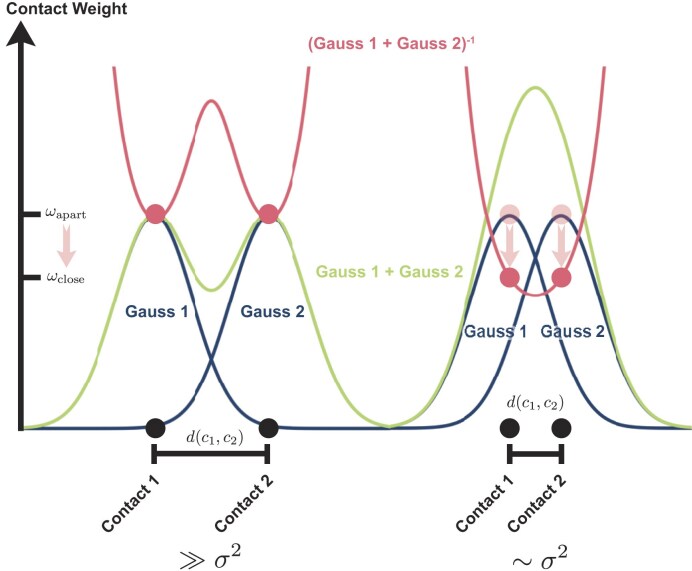
One-dimensional schematic of the Gaussian contact weighing. On the left are two contacts with a distance *d* much larger than σ^2^. On the right are the contacts closer together, and the overlap of the individual Gauss distributions (blue) is bigger. After summation (green) and inverting (red), the weights ω_close_ decrease compared with ω_apart_ for the contacts further away.

**Figure 3.**
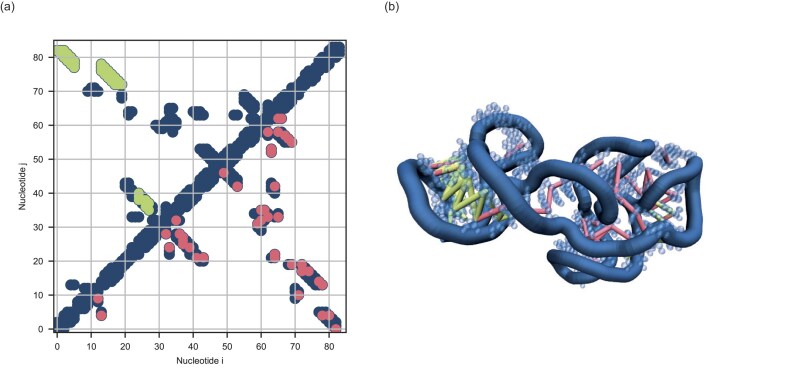
(**a**) Contact map of the cobalamin riboswitch aptamer domain (ID: 4frg). In dark blue is the native contact map as derived from the crystal structure. In green are shown a possible choice of restraints forming clusters and in red randomly distributed contacts. (**b**) The three-dimensional structure of the molecule 4frg from the test set $\mathfrak {D}$, determined experimentally. The coloured bonds represent the contacts belonging to (a).

**Figure 4.**
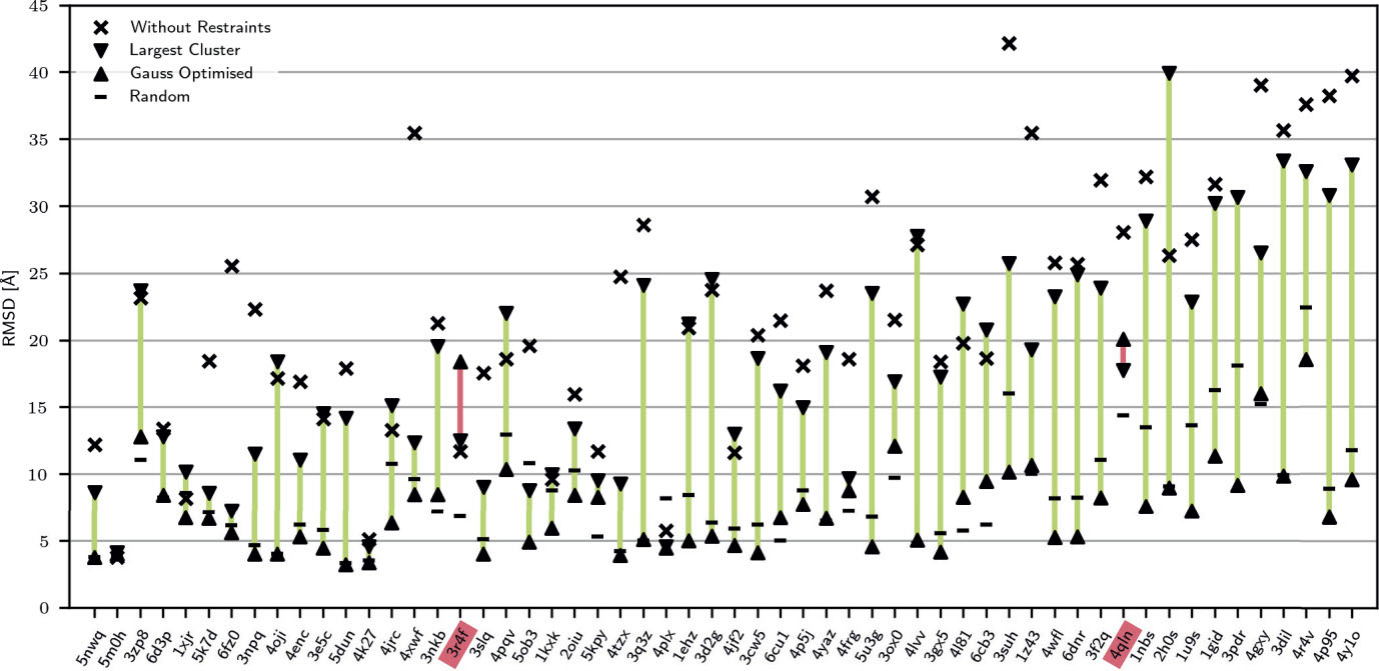
Complete visualization of the prediction quality for all molecules for different distributions of contacts. The molecules are sorted by size, and increase in size from left to right. The IDs from the PDB [38] are plotted on the *x*-axis. All distributions have been derived from the native contacts from experimentally determined structures. We used different methods for this: random, clustered, and Gaussian optimized. Simulations without any restraints are also shown for comparison.

## Materials and methods

In this section we give an overview of our test setup and the different computer experiments we ran on the test set to gain insight into the simulation quality in relation to different contact map topologies.

### Simulation software and test data

As simulation software, we use SimRNA [23], a software based on replica exchange Monte Carlo (REMC), with the standard configuration and 10 replicas per simulation. We use the representative of the largest cluster as the final structure. The contacts are added as restraints in the energy function. For the penalty term, see Supplementary Fig. S1. We have taken into account both short-range and long-range interactions (as proposed in [25]), but have ensured that theenergy cost remains constant above a certain threshold in orderto prevent the energy from being dominated by a single unfulfilled restraint.

The presented computer experiments were performed with a test subset $\mathfrak {D}$ of RNA families composed in Pucci *et al.* [35]. We had to sort out the structures that had a faulty PDB structure and thus made a comparison with our simulation results impossible. This effectively gave us 56 RNA families representing ncRNA molecules ranging in length from 41 to 496 residues. In the Application part, we use a different test set $\mathfrak {D}_{\mathrm{Val}}$. This was necessary because some of our original set $\mathfrak {D}$ were part of the training of the algorithms under investigation, and we would therefore suffer from data leakage. We are rebuilding the new validation set $\mathfrak {D}_{\mathrm{Val}}$ from scratch using special software called NucleoSeeker [37]. We apply the following criteria: firstly, the filtered structures must have been published after the training data of our AI algorithms (i.e. from 2023 onwards) and, secondly, they must show a significant deviation from already known structures (sequence similarity ≤80%). These strict guidelines are essential in order to draw meaningful conclusions and avoid data leakage. Unfortunately, these specifications result in a very limited validation set of only 20 structures. After creation of the corresponding MSA, most structures also have an effective family size of <5. In addition, some of the structures are bound or occur in a complex, which further complicates structure prediction. We removed one structure (7yga) because it represents a dynamic intermediate step in splicing and therefore cannot be meaningfully captured by static structure prediction. In this section we have also adapted our contact definition to the definition used in the original publications of the methods. Supplementary Tables S1 and S2 provide an overview of all families, representing molecules, their size, and the effective family size.

### Definition of Contact

In the course of our analysis, we use two different definitions to describe two residues of an RNA molecule as being in contact. In most of our computer experiments, we use the definition used in Pietal *et al.* [32], which is a fundamental work on contact maps for RNA molecules. Two residues are according to this definitiona contact if their N1/N9 nitrogen atoms are no more than 9.5 Å apart. The AI systems in the Application part were trained and published using a different definition. In their publications, the authors used 10 Å from the heavy atoms that are closest together. To keep the analysis consistent, we have therefore adjusted our definition in this section. It should be noted that whether nitrogen atoms (9.5 Å) or nearest heavy atoms (10 Å) as definition does not greatly influence the outcome, as shown and investigated by Pucci *et al.* [35].

### Distribution of contacts

The results of CoCoNet [30] suggest that the distribution of contacts has a direct influence on the prediction of the spatial structure of the RNA molecule with $L$ nucleotides. In order to investigate this influence, three distinct contact distributions are generated for each molecule within the test set $\mathfrak {D}$. The individual distributions are constructed by generating the contact map from the experimentally determined three-dimensional structure and selecting a subset of $L/2$ contacts from it. These $L/2$ contacts are selected in the following ways: (i) clustered; (ii) randomly; and (iii) Gaussian weighted. This gives us three different contact map topologies for each molecule.How well the three different topologies improve the simulation and whether the simulation benefits at all from this information are discussed in the Results and discussion.

The clustered contact maps are an extreme example of the way in which a CNN, for example, operates contact prediction. The probability of finding a contact is increased if there is already a contact in the immediate vicinity. We select our contacts from the complete contact map in which we have previously identified all clusters. We use a very fast calculation method for cluster definition and determination. Two contacts are in the same cluster if there is a path between the two contacts via other contacts. The method produces visually good results for the contact maps for molecules, whereby it is important not to include diagonal contacts and their neighbours. Advanced clustering algorithms such as k-means are therefore not necessary. After the clustering process, we randomly select contacts from the largest cluster until all contacts from that cluster have been selected, and then move on to the next smallest cluster using the same method. An example of such a selection is drawn in Fig. 3a.

The randomly distributed contacts represent the opposite extreme case. In this distribution, the individual restraints are randomly selected from the entire contact map. Although we know the complete contact map in advance in our computer experiments, this is precisely the goal in normal RNA structure prediction. Existing algorithms offer a larger number of contacts, but the confidence of the individual contacts decreases significantly. It is precisely the selection of a subset of contacts based on confidence that enables the algorithms to make good predictions. This means that it is not possible to select contacts from the initially unknown contact map, nor is it possible to randomly select possible contacts neglecting the confidence. What is needed is a measure that determines the degree of randomness. We could incorporate this measure into existing algorithms and thus achieve a complex prediction that takes into account the topology of the contact maps.

This measure, which determines randomness, should meet a few requirements.

- It should be possible to determine this without being aware of the unknown contact map.
- It should be continuous.
- It should be smaller for many contacts that are close together and larger for those that are further apart.
- Once a certain spread has been reached, the value should no longer change.

We introduced this measure with a Gaussian weighting of the individual contacts. Instead of valuing each contact equally, contacts that are close to each other are devalued. A one-dimensional schematic representation of this can be seen in Fig. 2. Two scenarios are depicted there: on the one hand, two contacts in the one-dimensional contact map that are further apart and, on the other hand, those that are close together. The influence of each contact on the neighbouring contact is modelled with a Gaussian function (blue curve) and the addition of all influences of the other contacts gives the weighting of the current contact (green curve). To devalue the individual weights instead of enhancing them, this weighting is inverted (red curve). Here, the value at the point of contact can be read directly from the curve and, the closer two contacts are to each other, the lower the value (hereafter referred to as the Gauss score). Generalized to two dimensions, we obtain the value $\nu _i$ for the addition of all Gaussian functions for each contact $i$ with position $\boldsymbol {r}_i$ on the contact map with all contacts $\mathcal {C}$:


(1) \begin{eqnarray*}
\nu _i = \sum _{j \in \mathcal {C}}\exp \left[-\frac{(\boldsymbol {r}_i - \boldsymbol {r}_j)^2}{\sigma ^2}\right]
\end{eqnarray*}

Each contact, which is surrounded by other contacts, now has a higher $\nu$ compared with isolated contacts. To devalue the contacts, we introduce the inverse $\omega _i = \nu _i^{-1}$ as weights. The total sum of weights is now reduced for a clustered contact selection and is at its maximum for widely dispersed contacts. The latter is closer to a random selection of contacts, so you can incorporate


(2) \begin{eqnarray*}
\Omega = \sum _{i \in \mathcal {C}} \omega _i
\end{eqnarray*}

as an additional optimization parameter in ML algorithms for the contact diversity. This parameter is a measure of the topology of the contact map. It can be used to evaluate existing algorithms, as we do in the Applications part, or to develop new algorithms that use it as an additional optimization parameter which we also show in Applications. This is also the advantage over randomly distributed maps, which, although they are maximally spread, have no quantitative measure that can be optimized in calculations. Even though the selection of $\Omega$ appears arbitrary, it is not. The Gaussian bell curve approach is a widely used method for modelling an exponential decline. In our model, we use it to represent the declining influence of contacts on each other. With the above argument, we have already covered all the requirements we previously set for such a measure. The summation and inversion serve to reduce the value to a measure that can be incorporated as an additional term in the loss function of an AI algorithm. In the Applications part, we do this for a state-of-the-art transformer model for contact map prediction of RNA molecules.

### Influence of false contacts

In reality, it is impossible to use only true positive contacts, since even the best current ML methods only achieve a PPV of ~80% [29]. In the next step, we introduce false contacts to our contact map. We therefore use randomly chosen contacts $\bar{\mathcal {C}}$ from the set of residue pairs which are not part of the native contacts. The remaining contacts, designated as set $\mathcal {C}$, are generated through a Gaussian optimization process, derived from the native contacts. The PPV decreases from 1 to $1-\lambda$, where $\lambda = |\bar{\mathcal {C}}|/|\mathcal {C}\cup \bar{\mathcal {C}}|$ is the fraction of false contacts in our selection (error rate). With the selection $\mathcal {C} \cup \bar{\mathcal {C}}$ we can simulate each molecule $\mathfrak {d}$ from our test set and calculate the $\mathrm{RMSD}(\mathfrak {d},\ \mathcal {C} \cup \bar{\mathcal {C}})$. To give a short overview of the quality, we introduced the beneficial fraction


(3) \begin{eqnarray*}
\xi (\lambda ) = \frac{1}{|\mathfrak {D}|}\sum _{\mathfrak {d}\in \mathfrak {D}} \Theta \left[ \mathrm{RMSD}(\mathfrak {d},\ \varnothing ) - \mathrm{RMSD}(\mathfrak {d},\ \mathcal {C} \cup \bar{\mathcal {C}}) \right]
\end{eqnarray*}

which shows the fraction of molecules for a given $\lambda$ with a lower RMSD with given restraints compared with the same molecule without any restraints. In order to stay as close as possible to reality, we have decided on Gaussian-optimized contacts for the true positives and random for the false positives. This should best represent the influence of the false positives.

### Applications

In order to analyse the individual influences and results on the structure prediction and the Gaussian score in practice, we looked at and compared three state-of-the-art methods for contact map creation. As a benchmark, we start with the DCA algorithm, which has a lower PPV but should have a diverse contact map topology. We used the implementation pyDCA with the mean field approximation (mfDCA). As a representative of a sparse learning algorithm (CNN) we take CoCoNet, which, as already mentioned in the Introduction, has extremely good PPV values but not much gain in structure prediction [35]. The third method uses the advanced AI algorithm Barnacle, which has been specially trained for contact map prediction. Barnacle uses a transformer model to create a prediction that is as accurate as possible. As an enhancement of these three methods, we completely retrained Barnacle and added the Gaussian score from Equation (2) as an additional term in the loss function. This additional term in the loss function was assigned a scalar weighting (we used −1 for proof of concept) and a variance σ^2^ of 4. We determined the scalar weighting so that we could see a significant increase in $\Omega$ during training, and we used the variance by analysing existing contact maps, i.e. the effects of clustering are well represented in $\Omega$. These two parameters are the only freely selectable constants. Other values for these two constants are certainly conceivable, but it would go beyond the scope of this study to carry out a detailed analysis of them as well. These two parameters can be considered well chosen if we see a decrease in PPV from the modified version to the generic version and an increase in $\Omega$ of the modified version during training. Here, a trade-off must be made between the extent to which the PPV is sacrificed for the topology. During our training of the modified Barnacle model, where we optimize the AI model parameter but not our chosen parameters, we saw, as expected, a decrease in PPV (from 0.46 to 0.38) compared with the unmodified (generic) version. When we looked at the Gaussian score separately for the training, we saw an increase (from first epoch 63.40 to last epoch 187.00). We can therefore be sure that we have retrained the system correctly. For all four methods, we compare the precision and the Gauss value and take a closer look at contact maps. We then run simulations using SimRNA for all contact maps created, allowing us to compare the RMSD for all methods and all molecules of $\mathfrak {D}_{\mathrm{Val}}$ at the end. In order to reproduce the previous results from the literature [29, 35], we also switch to a different contact definition. We count two residues $i,j$ as contact if, and only if, there is a pair of heavy atoms that are <10 Å apart.

## Results and discussion

### Distribution of contacts

We investigated the initial question of whether the influence of the distribution of contacts has an impact on the prediction quality by simulating different distributions of contacts. We investigated the three different distributions clustered, randomly, and Gaussian optimized as presented in the Materials and methods. After creating these contact maps, we can use standard statistics, such as Hopkins statistics, to examine whether our approach has actually led to a reduction in clustering. Indeed, the Hopkins value drops from 0.99 for the clustered contact maps to 0.91 for the random ones and to 0.87 for our Gaussian-optimized ones. Our method thus works as expected, and we can proceed with the structure prediction. We enter all our contact maps as restraints in SimRNA and calculate the RMSD after the simulation. The results are marked for all individual molecules into a common overview (see Fig. 4). For comparison, the simulation without restraints is also presented, and it is clear that almost without exception the addition of restraints improves the prediction quality. The distribution with the clustered contacts gives only a slight improvement in the prediction. However, the Gaussian-optimized distribution shows a significant improvement in the RMSD. Only two molecules (3r4f and 4qln, marked in red in Fig. 4) do not benefit from the Gaussian contact distribution. However, the quality of these molecules either is already in a very high range (4qln) or both clustered and Gaussian contacts are worse than without restraints (3r4f), which points to a more fundamental problem. We have provided the two corresponding contact maps in Supplementary Fig. S2. However, these do not reveal any anomalies. One possibility is that both molecules are dimers and that focusing on the largest cluster gives them an advantage in folding. Nevertheless, folding using SimRNA is also a stochastic process that can lead to individual outliers. For all other molecules, the Gaussian-optimized distributions are in the range of randomly selected contacts. We can draw two conclusions from these results. Firstly, our hypothesis that a more diverse contact map contributes significantly to prediction quality is correct. Secondly, our Gaussian weighting $\Omega$ (see Equation 2) is a useful property of a contact map to evaluate the diversity or randomness of the same. Before we turn to the results of the specific applications, we want to analyse the influence of false contacts.

### Influence of false contacts

**Figure 5.**
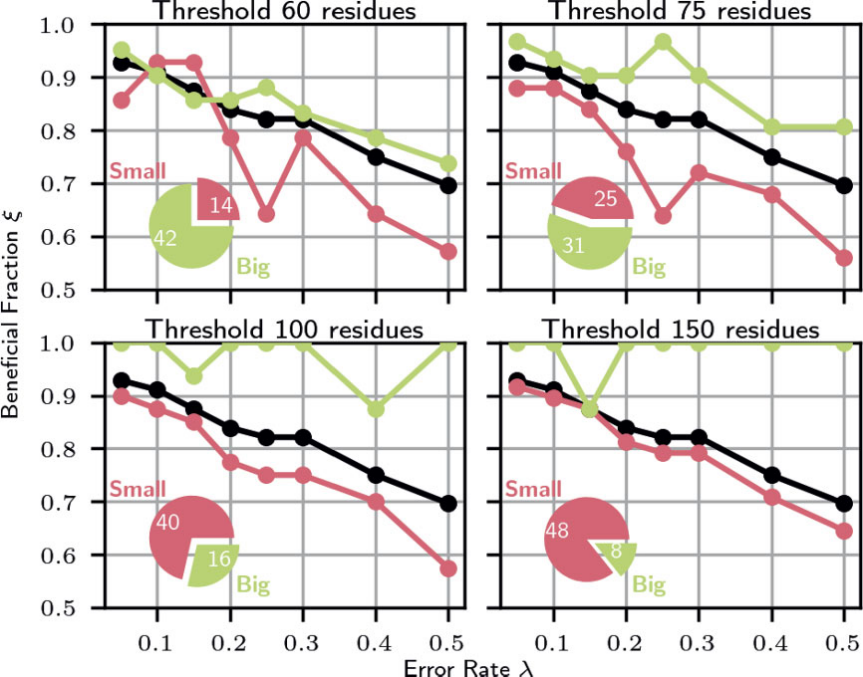
Illustrations of the beneficial fraction. In black is the whole test set for comparison, in green the larger molecules above the specific threshold, and in red the smaller portion with fewer or an equal number of residues. The pie chart is a visual representation of the ratio and number of molecules for the individual subsets. This means, for example, that with a threshold of 60 residues, 14 molecules in our test set are shorter and 42 molecules are longer. We have illustrated this binary division for different thresholds.

Assuming we include false contacts in our contact maps, the false contacts should also lead to a worse prediction. In Fig. 5, we used the beneficial fraction from Equation 3 to analyse the PPV at which it is better not to specify any restraints for the simulation of an unknown molecule.

The diagram demonstrates that even for a high error rate $\lambda$ of 0.5, 70% of the molecules still benefit from the restraints. We can delve deeper into our test set by dividing it into two groups: larger molecules and smaller molecules. This is done by setting a threshold. We use the randomly selected values of 60, 75, 100, and 150 residues as the threshold. All molecules smaller than or equal to this value show a lower beneficial fraction than the larger ones in the analysis, with the exception of the very small molecules at very low error rates. However, this may also be due to the statistical significance of the very small sample. We see that the larger molecules in particular benefit from the additional restraints. For instance, the eight largest molecules profit exclusively from the inclusion of restraints, even though half of all contacts are false contacts. This admittedly very superficial analysis neither provides explanations nor does it say anything about specific molecules. However, it shows that even with the most unfavourable choice of false contacts, their inclusion statistically leads to a better prediction. It is therefore highly advisable not to use a pure simulation, especially for larger molecules, but to perform an evolutionary analysis beforehand. Ideally, this evolutionary analysis should have as diverse a contact distribution as possible, as shown in the previous section. A detailed list of all individual molecules can be found in Supplementary Table S1.

### Applications

We have seen in the previous section that the inclusion of evolutionary data is always advantageous, even if the false contacts are maximally unfavourably located. The first section has already shown that the topology of the contact map has a decisive influence on the quality of the structure prediction. Finally, let us look again at familiar ML algorithms CoCoNet [35] and Barnacle [29] and try to explain the lack of structure prediction quality by including the topology of the contact map.

**Figure 6.**
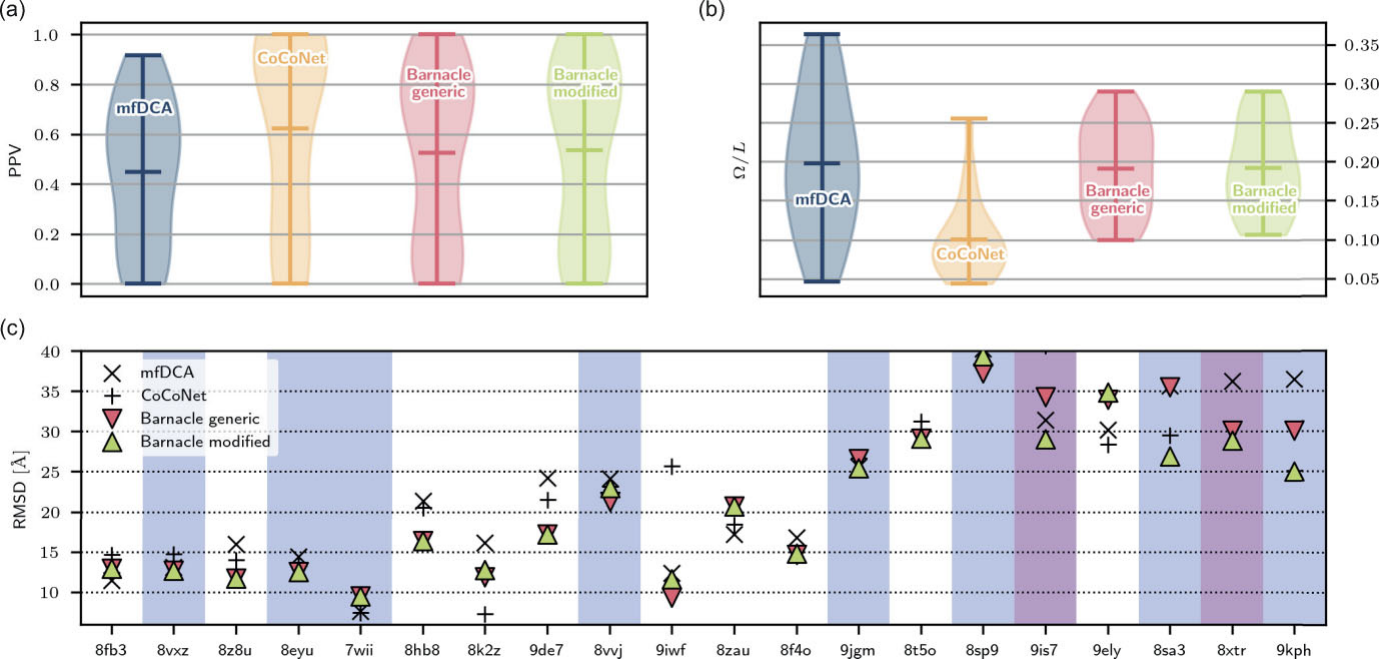
(**a** and **b**) Study of the individual quantities (PPV and $\Omega /L$) of the predictions on the test set $\mathfrak {D}_{\mathrm{Val}}$. Despite Barnacle’s retraining, $\Omega$ does not increase for our validation set. This is probably due to the poor quality of the test set. (**c**) Representation of the RMSD for all molecules in ascending length $L$ of $\mathfrak {D}_{\mathrm{Val}}$. The restraints for the simulation were created using different contact maps (mfDCA, CoCoNet, Barnacle generic, and Barnacle modified). Bound molecules are marked in light blue and larger complexes in purple. The interpretation of the values is given in the main text. A listing of the validation set and the analysis of all individual molecules is given in the Supplementary Table S2 and Supplementary Figs S3, S4 and S5.

First of all, let us get a rough overview of the complete validation set (all contact maps are depicted in Supplementary Figs S3, S4, and S5). Figure 6a shows the PPV as an established quality value, and Fig. 6b the Gaussian value introduced by us, normalized to the size of the molecule, for quantifying the topology. As assumed in the Introduction, it is already apparent at first glance that the ML algorithms, especially CoCoNet, have a more clustered topology compared with the DCA algorithm. However, you can also see a difference between the two ML algorithms. The shallow algorithm CoCoNet leads to very poor Gauss values. In contrast, the algorithm Barnacle, which is based on a transformer architecture, provides a significantly higher Gauss value. If we now consider the differences between the modified Barnacle and the original, there is no noticeable difference in PPV. In terms of the Gaussian score, the modified algorithm even shows slightly lower values. These are initial indications that our dataset could be problematic, as the training of Barnacle showed significantly increasing Gaussian scores. In the next step, we created the structure prediction for all four methods using SimRNA and evaluated the RMSD. Figure 6c shows all values for all molecules. All molecules with a length of <100 residues [*L*(9jgm)=106] show no differences whatsoever between the generic and modified Barnacle versions. However, differences become apparent for larger molecules. Some molecules seem to benefit from the Barnacle modification; at the very least, it does not make the prediction any worse. However, the figure also shows that many of the molecules are in a bound state or form complexes. This makes structure prediction using physics-based methods such as SimRNA very difficult without the complex partner. It is therefore all the more surprising that, despite all these adversities, we were able to observe a significant improvement in RMSD for some larger molecules, even though, as already described, the PPV deteriorated during training. At this point, it should be noted once again that, at the present time, it was not possible to generate a higher quality validation set $\mathfrak {D}_{\mathrm{Val}}$, as this would inevitably result in data leakage. This would dilute the significance of the study or render it invalid. The lack of data leading to this suboptimal validation set also emphasizes the importance of including the contact map topology in the design of AI algorithms for structure prediction. Really good results and unnecessary detours can only be achieved with the inclusion of a good topology and not with pure PPV-orientated training. In the already very successful algorithms for protein structure prediction, consideration of the topology may already be priced in by the large amounts of training data, but these amounts do not exist for RNA, and the use of such knowledge is elementary for a good prediction.

## Conclusion

We investigated whether the topology of contact maps, which are output by modern AI algorithms for RNA structure prediction, has an influence on the prediction quality. To answer this question, we created artificial contact maps and used a large test set to show that the topology has an important influence. To quantify the topology, we also introduced a new measure, the Gaussian score. In the last section, we applied the measures PPV and Gauss to a smaller validation set. We had to build this validation set from scratch, and we included all RNA structures published after the AI algorithm was trained to prevent data leakage. Then we carried out a structure prediction for all the methods examined. At this point, however, it should also be said that the validation set in particular is of poor quality. In some cases, the underlying MSA consisted of only a few entries. Therefore, the absolute values in particular should be treated with caution. The individual contact maps are also shown in Supplementary Figs S3, S4, and S5, and in some of them you can see a completely inaccurate prediction of contacts. Another limitation of our analysis is the dependence on the simulation software SimRNA. However, the aim was not to achieve absolute numbers and the best possible prediction, but rather to compare different starting conditions as independently as possible. This is solved very well with SimRNA, since the simulation follows a physical force field, and not learned motifs, as with trained AI algorithms. For RNA, we have the big problem of limited datasets for training large language models, so an approach such as AlphaFold is not yet feasible. Although AlphaFold 3 [31] has the option of RNA structure prediction, initial tests show that its quality does not match the prediction quality of proteins [39].

It is therefore of great importance to have clues for a good structure prediction in advance, in order to compensate for the lack of training data. The incorporation of topology is one such clue, which is of particular importance for architectures such as CNNs. In the future design of AI algorithms for structure prediction, on the one hand, such insights can be integrated and provide direction for the network structure. On the other hand, the Gaussian score can also be used directly as a candidate term in the loss function. This allows the algorithm to directly train an optimized topology for ideally matched contact maps for structure prediction.

Lastly, DCA identifies co-evolutionary signals within protein or RNA families, even to the extent of judging fitness landscapes [40, 41]. The Gaussian score provides a quantitative measure of the extent to which evolutionary constraints act as delocalized across the contact map. Thus, it can be employed to assess whether evolutionary processes preferentially stabilize localized clusters of contacts or exert their influence in a more dispersed manner across distinct regions of the contact map.

## Supplementary Material

gkaf1370_Supplemental_File

## Data Availability

The data underlying this article are available via Zenodo https://doi.org/10.5281/zenodo.16964019.

## Notes

Present address: Scientific Computing Centre, Karlsruhe Institute of Technology, D-76344 Eggenstein-Leopoldshafen, Germany
