# Supplementary material for: Influence of contact map topology on RNA structure prediction
Source: Nucleic Acids Res. 2025 Dec 17;53(22):gkaf1370. doi: 10.1093/nar/gkaf1370 (PMC12709182; doi:10.1093/nar/gkaf1370)
Supplement: gkaf1370_Supplemental_File [file gkaf1370_supplemental_file.pdf]

Influence of Contact Map Topology on RNA Structure Prediction - Supplementary Information

Christian Faber[1], Utkarsh Upadhyay[1], Oskar Taubert[2], Alexander Schug [1,3,*]

November 5, 2025

1 Software and Data

All software scripts, data and instructions how to use the code are stored in the following Git repository: https://github.com/KIT-MBS/contactmaps

2 Contact Definition

In the main part of our work, we used the following contact definition:

> *Two nucleotides i and j are considered to be in contact if their nitrogen atoms are within 9.5Å of each other.*

In the Applications section, however, we have adapted our contact definition to that of the original publication of the various methods. It reads:

> *Two nucleotides i and j are considered to be in contact if the nearest pair of atoms (a, b), where a is a heavy atom of nucleotide i and b a heavy atom of nucleotide j are less than 10Å apart.*

3 Penalty Function SimRNA

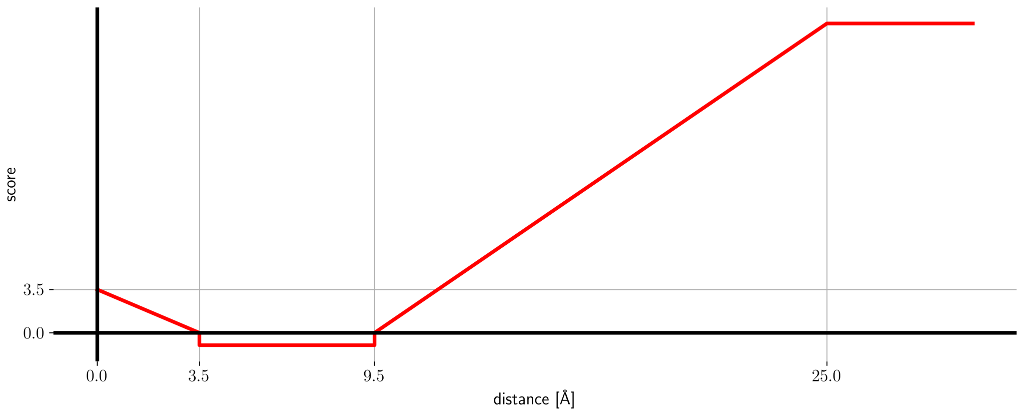

Supplementary figure 1: Each potential contact is weighted with this function in SimRNA.

4 Datasets

4.1 Test Set $\mathfrak{D}$

Supplementary table 1: Dataset for the main part of the work.

PDB	Family	L	M_{eff}
1ehz	RF00005_1	76	461.0
1gid	RF00028	159	799.3
1kxk	RF00029	70	51.0
1nbs	RF00011	120	157.6
1u9s	RF00010	155	1285.3
1xjr	RF00164	47	3.3
1z43	RF00169	101	281.5
2h0s	RF00234	123	603.7
2oiu	RF03017	71	2.0
3cw5	RF00005_2	77	705.0
3d2g	RF00059	77	1241.9
3dil	RF00168	173	1455.5
3e5c	RF01767	53	13.6
3f2q	RF00050	108	104.8
3gx5	RF00162	94	322.1
3nkb	RF02682	64	41.0
3npq	RF01057	51	10.5
3ox0	RF00504	87	847.6
3pdr	RF00380	161	119.6
3q3z	RF01786	75	372.1
3r4f	RF00044	66	2.0
3slq	RF01510	67	6.6
3suh	RF01831_2	101	198.5
3zp8	RF00163	43	59.0
4enc	RF01734	52	221.1
4frg	RF01689	84	62.8
4gxy	RF00174	162	6987.2
4jf2	RF01054	77	14.6
4jrc	RF02447	57	6.8
4k27	RF02695	55	32.0
4l81	RF01725	96	211.0
4lvv	RF01831_1	89	187.7
4oji	RF02684	52	4.7
4p5j	RF00233	84	16.2
4p95	RF01807	189	3.2
4plx	RF02266	76	2.0
4pqv	RF01415	68	4.9
4qln	RF00379	117	998.9
4r4v	RF02927	186	2.0
4tzx	RF00167	71	458.9
4wfl	RF01854	106	177.0
4xwf	RF01750	64	130.7
4y1o	RF02001_2	258	248.3
4yaz	RF01051	84	583.4
5dun	RF00921	54	2.0
5k7d	RF02679	47	32.4
5kpy	RF01982	71	2.0
5m0h	RF00606	42	2.0
5nwq	RF01763	41	5.1
5ob3	RF01300	69	2.0
5u3g	RF00442_1	85	33.9
6cb3	RF00080	99	409.5
6cu1	RF02553	80	91.4
6d3p	RF02888	45	1.0
6dnr	RF00442_2	107	137.8
6fz0	RF01826	48	2.8

4.2 Validation Set $\mathfrak{D}_{\text{Val}}$

Supplementary table 2: Dataset for the application section.

PDB	Family	L	M_{eff}
7wii	RF02977	50	2.6
7yga	RF00028	393	9.0
8eyu	RF00168	49	3.1
8f4o	RF00059	83	67.5
8fb3	RF00522	34	5.8
8hb8	RF01079	55	2.0
8k2z	RF01684	57	4.6
8sa3	RF00174	210	294.5
8sp9	RF00386	153	4.0
8t5o	RF00163	124	4.0
8vvj	RF01750	64	53.5
8vxz	RF03493	36	2.0
8xtr	RF03300	219	2.0
8z8u	RF01136	41	2.0
8zau	RF00419	69	7.9
9de7	RF00250	58	1.9
9ely	RF03072	205	51.8
9is7	RF03013	153	9.1
9iwf	RF00167	69	44.7
9jgm	RF00176	105	2.0
9kph	RF02933	255	14.8

5 Contact Maps

5.1 Distribution of Contacts

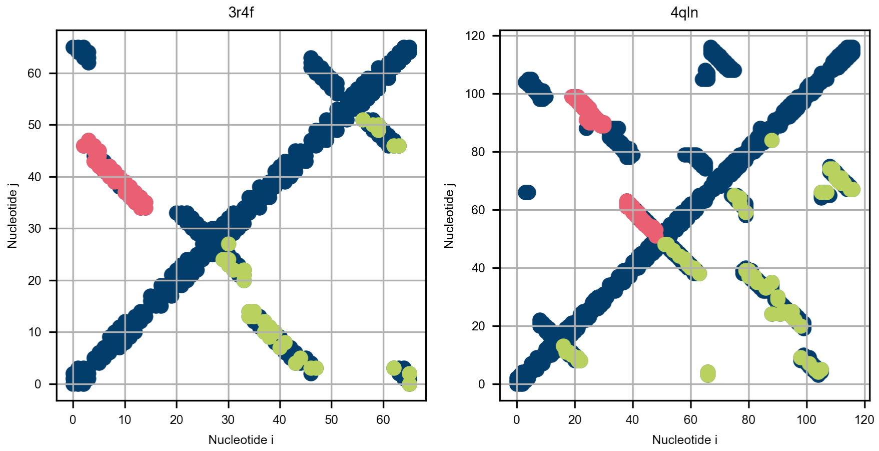

Supplementary figure 2: Contact maps for the two outliers, i.e. the prediction is better for the clustered restraints (red) compared to Gauss optimised (green).

5.2 Applications

All the contact maps from the application section:

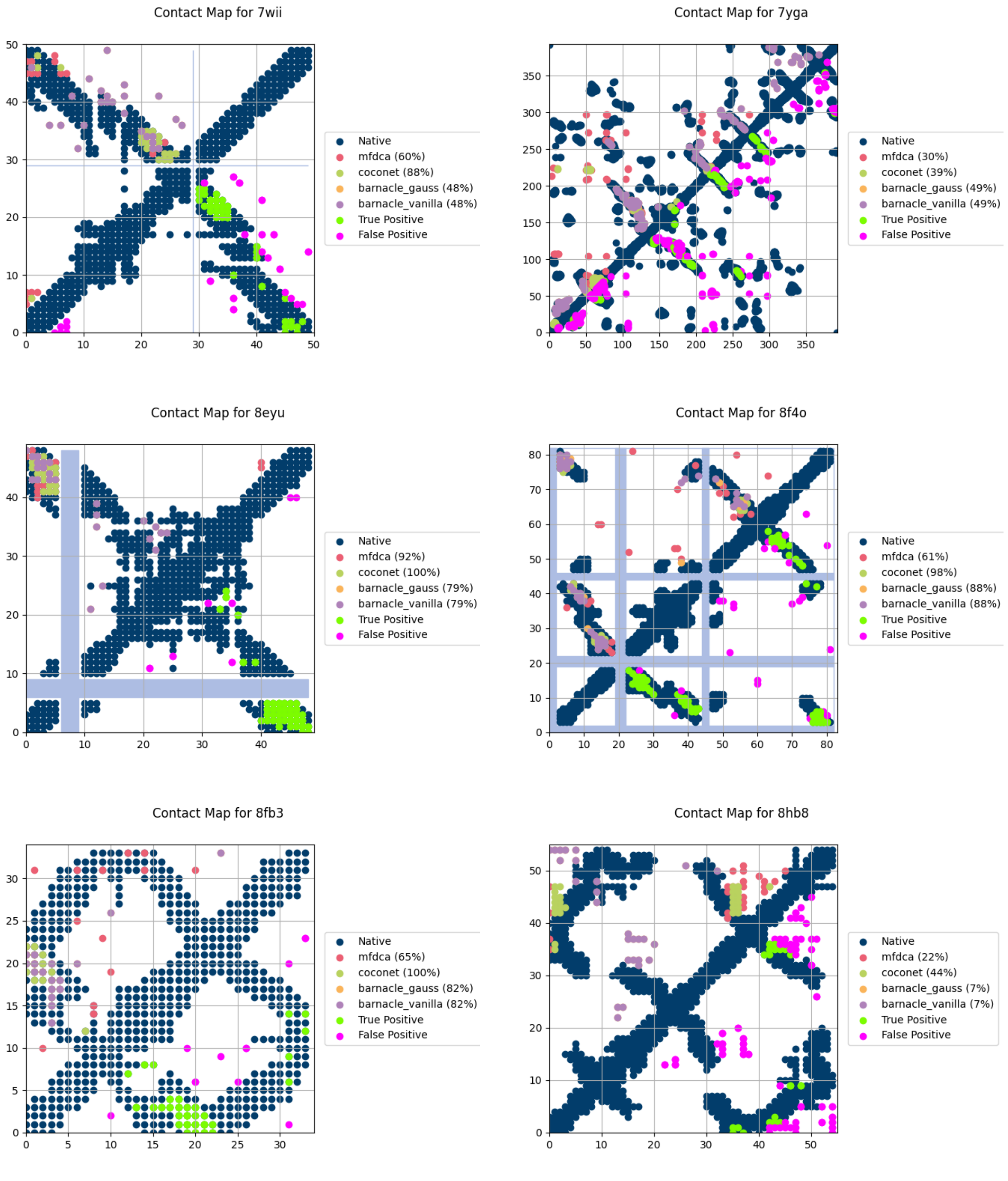

Supplementary figure 3: Contact Maps

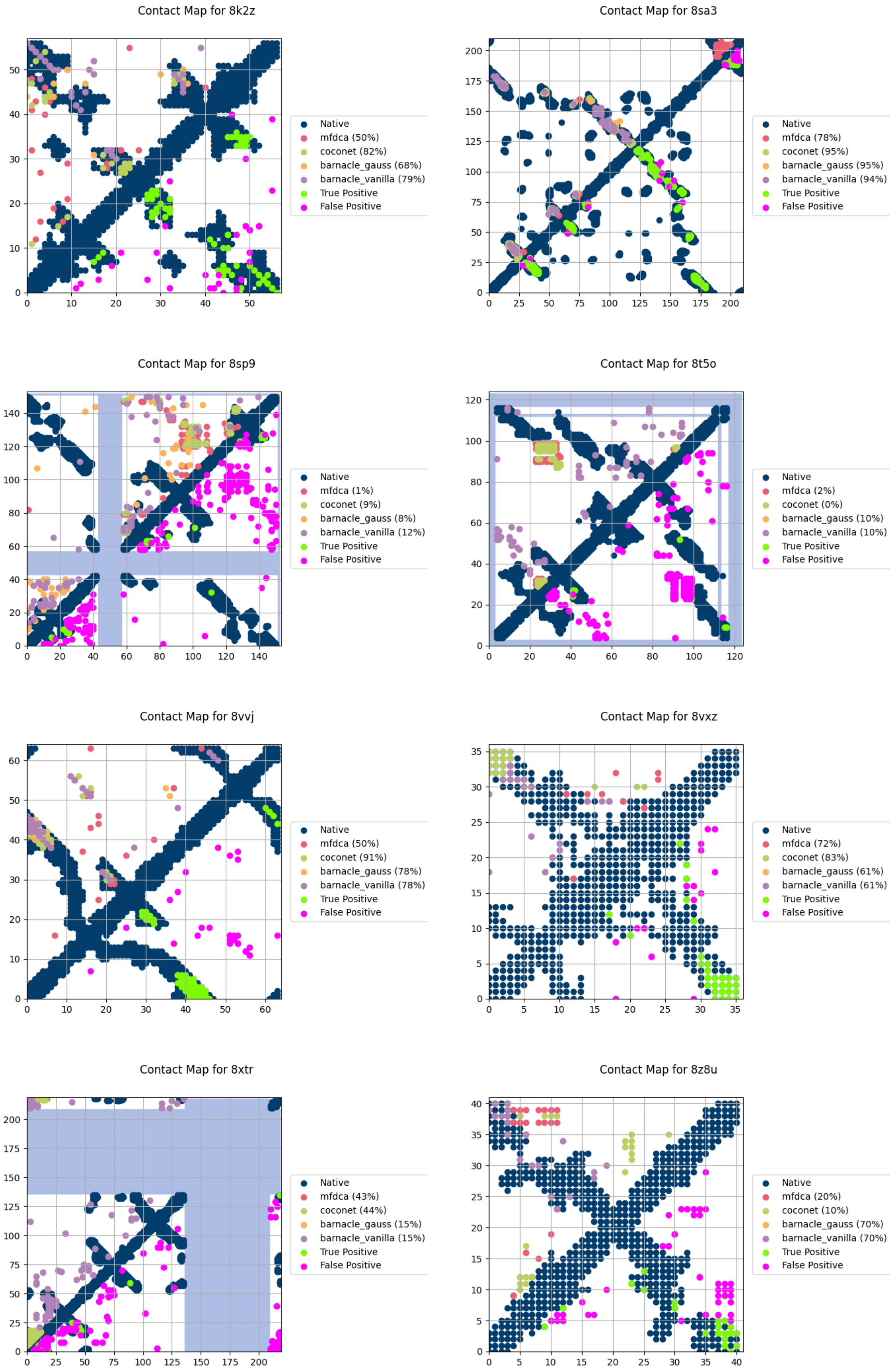

Supplementary figure 4: Contact Maps

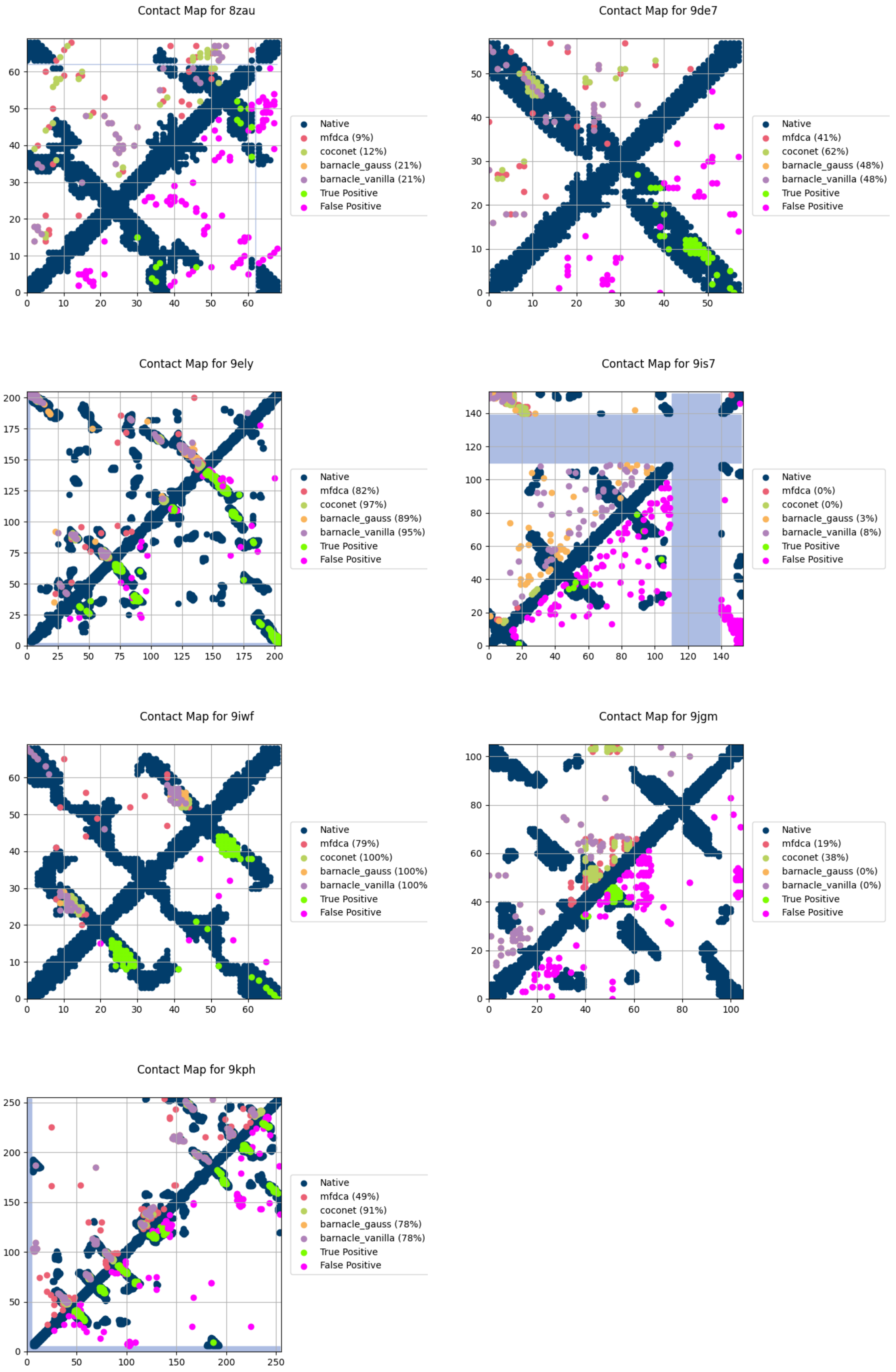

Supplementary figure 5: Contact Maps
